# Virus Dynamics Are Influenced by Season, Tides and Advective Transport in Intertidal, Permeable Sediments

**DOI:** 10.3389/fmicb.2017.02526

**Published:** 2017-12-18

**Authors:** Verona Vandieken, Lara Sabelhaus, Tim Engelhardt

**Affiliations:** Paleomicrobiology, Institute for Chemistry and Biology of the Marine Environment, University of Oldenburg, Oldenburg, Germany

**Keywords:** virus production, cell production, intertidal sands, advection, oxygen consumption, flow-through reactors

## Abstract

Sandy surface sediments of tidal flats exhibit high microbial activity due to the fast and deep-reaching transport of oxygen and nutrients by porewater advection. On the other hand during low tide, limited transport results in nutrient and oxygen depletion concomitant to the accumulation of microbial metabolites. This study represents the first attempt to use flow-through reactors to investigate virus production, virus transport and the impact of tides and season in permeable sediments. The reactors were filled with intertidal sands of two sites (North beach site and backbarrier sand flat of Spiekeroog island in the German Wadden Sea) to best simulate advective porewater transport through the sediments. Virus and cell release along with oxygen consumption were measured in the effluents of reactors during continuous flow of water through the sediments as well as in tidal simulation experiments where alternating cycles with and without water flow (each for 6 h) were operated. The results showed net rates of virus production (0.3–13.2 × 10^6^ viruses cm^−3^ h^−1^) and prokaryotic cell production (0.3–10.0 × 10^5^ cells cm^−3^ h^−1^) as well as oxygen consumption rates (56–737 μmol l^−1^ h^−1^) to be linearly correlated reflecting differences in activity, season and location of the sediments. Calculations show that total virus turnover was fast with 2 to 4 days, whereas virus-mediated cell turnover was calculated to range between 5–13 or 33–91 days depending on the assumed burst sizes (number of viruses released upon cell lysis) of 14 or 100 viruses, respectively. During the experiments, the homogenized sediments in the reactors became vertically structured with decreasing microbial activities and increasing impact of viruses on prokaryotic mortality with depth. Tidal simulation clearly showed a strong accumulation of viruses and cells in the top sections of the reactors when the flow was halted indicating a consistently high virus production during low tide. In conclusion, cell lysis products due to virus production may fuel microbial communities in the absence of advection-driven nutrient input, but are eventually washed off the surface sediment during high tide and being transported into deeper sediment layers or into the water column together with the produced viruses.

## Introduction

Approximately 20% of the primary production in the oceans takes place at the continental shelves (Jahnke, 2010). Due to their low water depth (on average 65 m), a large fraction of the primary production reaches the seafloor and becomes available for benthic mineralization. Sandy sediments cover almost 50% of the shelves' seafloor (Hall, 2002), and through their large pore spaces water is transported by advection (Huettel et al., 2014). Advective transport is driven by hydraulic pressure gradients and is orders of magnitude faster than diffusion. Thereby, it facilitates the deep penetration of particulate and dissolved organic carbon as well as oxygen and sulfate into the sediment fueling microbial degradation processes (Huettel et al., 2014).

In North Sea seafloor sediments, which are predominantly covered with sands (Ahmerkamp, 2016), the pressure gradients responsible for porewater advection mostly result from tidal pumping and wave action (Jansen et al., 2009). During low tide, intertidal sand flats and beaches fall dry, and in the absence of advection, oxygen is rapidly depleted and metabolic products accumulate (Jansen et al., 2009). During high tide, these degradation products can be flushed from the sediments by advective “skin circulation,” which pumps water through the surface layer with deep penetration of solutes like oxygen (Billerbeck et al., 2006). These processes result in very dynamic redox conditions with rapid spatial and temporal changes in the order of cm and minutes to hours, respectively. Nutrients are rapidly supplied from the bottom water into the surface of intertidal sands (Huettel et al., 2014).

Organic mineralization rates and prokaryotic abundance of up to 10^9^ cells cm^−3^ are high in coastal sands and comparable to diffusion-controlled, muddy sediments (Huettel et al., 2014). The bacterial community composition of sandy sediments is different to those of the bottom waters and muddy sediments and apparently is influenced by differences in permeability (Probandt et al., 2017). However, virus dynamics have not been considered or investigated for these sediments.

Due to their small size, viruses can be transported by advective flow through permeable sands. The transport of viruses can be halted by sorption to sediment grains which has been shown to depend strongly on size and surface characteristic of the respective virus but also on the water composition and surface characteristics of the sand (Bales et al., 1995; Dowd et al., 1998; Ghanem et al., 2016). Virus numbers in marine sediments range from 10^3^ to 10^10^ viruses cm^−3^ (Carreira et al., 2013; Engelhardt et al., 2014; Yanagawa et al., 2014). Their abundance originates from transport, production and decay within the sediments. In marine surface sediments, viruses are constantly produced at rates of 10^6^ and 10^8^ viruses cm^−3^ h^−1^ and their production is correlated to prokaryotic activity and carbon production (Hewson and Fuhrman, 2003; Glud and Middelboe, 2004; Middelboe and Glud, 2006; Middelboe et al., 2006; Danovaro et al., 2008a; Siem-Jørgensen et al., 2008; Montanié et al., 2015). Thus, virus turnover times in sediments was determined to range between 1 day and 1 month (Glud and Middelboe, 2004; Middelboe et al., 2006; Siem-Jørgensen et al., 2008). Due to the impact of viruses lysis on prokaryotic mortality, between 2 to 336% of the prokaryotic standing stock are removed per day (Hewson and Fuhrman, 2003; Glud and Middelboe, 2004; Siem-Jørgensen et al., 2008), which corresponds to 12 to 138% of prokaryotic net production (Mei and Danovaro, 2004; Middelboe et al., 2006; Danovaro et al., 2008b; Montanié et al., 2015). Overall, viruses can have a considerable impact on the benthic microbial community, e.g., by shaping the community composition and affecting biogeochemical cycling via the viral shunt (Proctor and Fuhrman, 1990; Steward et al., 1996; Suttle, 2007; Danovaro et al., 2008b).

Here, we used for the first time flow-through reactors to investigate virus dynamics and transport of viruses in permeable, intertidal sediments. The reactors were filled with sediment from two intertidal sands and water was pumped through to simulate advective porewater transport. Flow-through reactors allowed for frequent monitoring of virus and cell numbers in the effluents as well as measuring oxygen consumption as proxy for microbial activity. Experiments were conducted with continuous water flow through the sediments as well as in tidal simulation experiments with alternating cycles with and without water flow (each for 6 h). With this, we aimed to estimate virus and cell production rates including the influence of season and tidal cycles. We hypothesized a dependency of virus activity and abundance on prokaryotic activity and a dynamic interplay between viruses and host cells that is influenced by advection-driven transport during tidal cycles.

## Materials and methods

### Sampling sites

**Figure 1:**
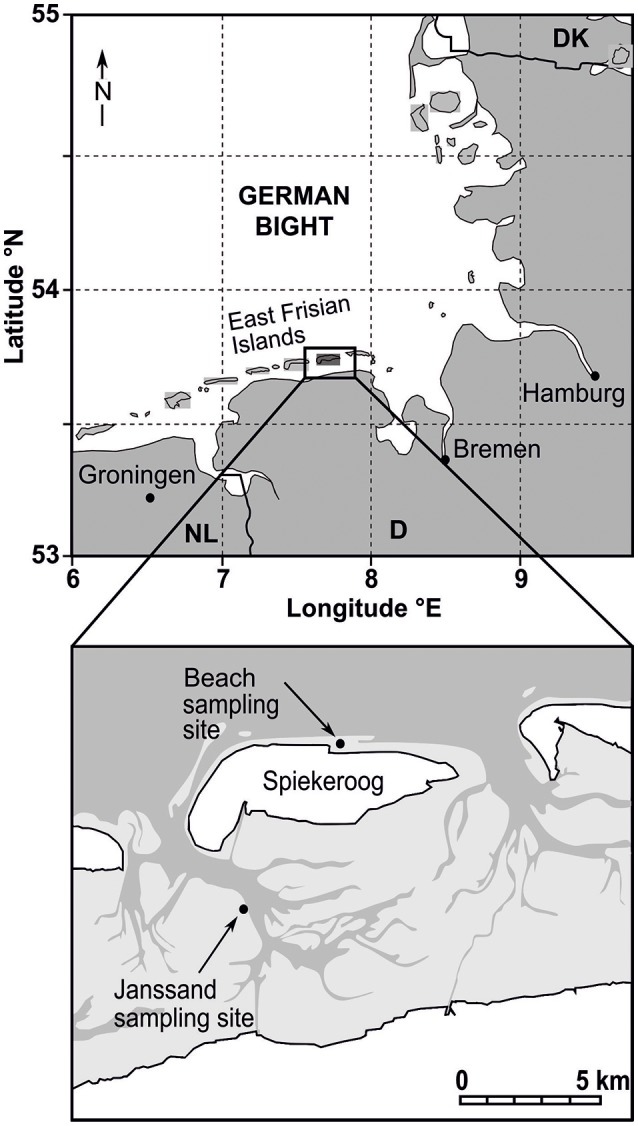
Map of sampling sites beach of Spiekeroog island and Janssand tidal flat adapted from Beck et al. (2017).

Surface sediment samples were collected from the Janssand sand flat in June 2016, July 2016, February 2017 and August 2017 and additional samples from the Spiekeroog beach site in November 2016 (Figure 1). Site Janssand (53° 44,183′ N; 007° 41,904′ E) is an intertidal sand flat with a mean tidal range of 2.6 m located in the backbarrier area of Spiekeroog island and has been intensively investigated with respect to porewater transport and biogeochemical processes (e.g., Billerbeck et al., 2006; Beck et al., 2008a; Røy et al., 2008; Jansen et al., 2009). During high tide, Janssand is covered by 1.5 to 2 m of water, while the sand flat is exposed for 6 to 8 h during low tide (Billerbeck et al., 2006). During exposure, the sediment remains water saturated, and air does not enter the pore spaces (Røy et al., 2008). Oxygen penetration differs depending on the tide cycle and wave action and can be absent or a few mm deep during low tide and up to several cm deep during high tide (Jansen et al., 2009). Porewater can be transported down to 50 cm depth during low tide due to hydraulic gradients, which result in a horizontal transport of water from the middle of the sand flat to the low water line, where the water runs out (Billerbeck et al., 2006; Røy et al., 2008; Jansen et al., 2009). The northern beach site of Spiekeroog is facing the open North Sea of Germany and is affected by 2.7 m mean tidal range (Beck et al., 2017). Both sediments were composed of fine and medium sand grains with sizes of 63 to 630 μm, which allow for porewater advection.

Seawater for the flow through the reactors was collected each time off site Janssand (also for the experiment with the beach sediment) and stored at 4°C in the dark. The water temperatures were 17°C in June 2016, 20°C in July 2016, 7°C in November 2016, 2°C in February 2017 and 19°C in August 2017.

### Experimental setup and sampling of the flow-through reactors

The seawater was filtered through 0.45 μm and 0.2 μm filters and additionally autoclaved either before or after 0.2 μm filtration. A single 10 l-reservoir was covered with a black cloth to keep it dark and was regularly exchanged. During the experiments in February and August 2017, the seawater in the reservoir was additionally bubbled with air. Virus and cell counts in the reservoir were monitored in detail only for the experiment in February 2017. Virus numbers ranged between 8 × 10^3^ and 8 × 10^6^ viruses ml^−1^ and cell numbers between 4 × 10^2^ and 4 × 10^6^ cells ml^−1^, because in some occasion cell growth occurred during water storage after the autoclaving and filtering of the seawater.

Flow-through reactors made of acrylic glass with a height of 17 cm and a diameter of 4.2 cm were filled with seawater to which homogenized surface sediment was slowly added avoiding the trapping of air bubbles (Supplementary Figure S1). The flow-through reactors were closed with a piston-like lid, which had two O-rings attached, leaving water of several cm heights above the sediment. The lids contained a small tube as an inflow port in the center, which was connected by 1.8 mm silicon or iso-versinic tubing via peristaltic pumps (pump tubing 1.8 mm tygon) to the seawater reservoir. The bottom of the flow-through reactors was connected to an outflow port. Sediment discharge was prevented by a plankton mesh (10 μm) placed on the bottom of the reactors. The reactors were covered with black cloths. Seawater was pumped from the top to the bottom of the flow-through reactors at a flow rate of 1 ml min^−1^. For reasons of practicability and availability of facilities, the flow-through reactors and the seawater were kept at 15°C in the dark for all experiments, which represents the annual average temperature.

The four flow-through reactors were filled with sediment to different heights for each experiment. In June 2016, the sediment lengths in the four reactors were 14.5 cm, in July 2016 1.9–2 cm and in November 2016 for the beach sediment 13.1–13.8 cm. In February and August 2017 different sediment heights were chosen for the four reactors: 3.4, 7.1, 11.5, and 11.6 cm in February and 1.7, 3.2, 4.6, and 5.5 cm in August. Varying sediment volumes were adjusted for differences in microbial activity and to ensure that the porewater did not become oxygen-depleted in the effluents of the reactors. Oxygen in the effluent was prerequisite to calculate oxygen consumption rates as a proxy for prokaryotic activity. Further, different sediment column lengths in February and August allowed the calculations of virus and cell production as well as oxygen consumption rates in different sediment sections by assuming that the short column was representative for the same sediment volume at the top of both, the medium and long columns, while the medium column was representative for the same volume at the top of the long column.

Initially, water was pumped continuously through the reactors for 5 to 7 days, after which tidal simulation cycles were run 1 to 12 times depending on the experiment, followed by 0.5 to 8 days with continuous water flow. Experiments with the reactors were in total run for 17 days in June 2016, 9 days in July 2016, 12 days in November 2016, 16 days in February 2017 and 8 days in August 2017. Tidal cycles were simulated in the reactors by 6 h of water flow followed by 6 h without water flow. For each experiment, one or two reactors were excluded for tidal simulation (none in August 2017), instead in these control reactors water was continuously pumped through the sediment. With the beach sediment, one reactor at the end of the experiment was used as a dead control. For this, seawater with 2% formaldehyde was pumped for 30 h through the reactor.

Samples for virus and cells counts in the effluent were taken twice a day during continuous flow. For tidal simulation experiments, selected tidal cycles were sampled in high frequency directly after the restart of the water flow. For enumeration of viruses and cells in the effluents, 1 ml of effluent was collected from the outflow port into a reaction vessel, fixed with 20 μl of 25% glutardialdehyde and frozen at −80°C. Similarly, samples from the reservoir were taken regularly. For oxygen measurements in the effluent, an oxygen flow-through cell with an integrated contactless optical oxygen sensor (Pyro Science GmbH, Aachen, Germany) was attached to the outflow port. Additionally, oxygen concentration of the seawater reservoir was regularly measured with the flow-through cells attached behind the peristatic pump.

For enumeration of viruses and cells in the sediment, samples were collected from homogenized sediments before the flow-through reactors were filled as well as at the end of each experiment from the reactors. For cell counts, 0.5 cm^3^ of sediment was fixed with 1 ml of 4% formaldehyde and stored at 4°C. For virus counts, 1 cm^3^ of non-fixed sediment was immediately frozen at −80°C. Porosity was calculated from wet weights and dry sediment densities in triplicates.

### Virus and cell counts of sediment and effluent samples

For the enumeration of prokaryotic cells from formaldehyde-fixed sediment, samples (0.5 cm^3^) were centrifuged for 3 min at 13,000 × g and the supernatant was discarded. Cells were extracted with 1 ml 5 mM sodium pyrophosphate buffer for 15 min in the dark, followed by sonication for 3 min. Sediment particles were allowed to settle for 20 s and the supernatant was transferred into a new tube. Similarly, the sediment was washed 6–8 times with 1 ml PBS and the supernatants were pooled. The extract was filtered onto a 0.2 μm pore size filter (25 mm, Nuclepore track-etched membranes, Whatman, Buckinghamshire, England) and stained with SYBR Green I. Cells were counted with an epifluorescence microscope Olympus bx51 within 10 to 20 randomly chosen counting fields and at least 400 cells counted.

Extraction of viruses from sediment was performed according to Danovaro and Middelboe (2010). In brief, 1 cm3 of sediment sample was extracted with 4.5 ml 5 mM sodium pyrophosphate buffer for 15 min at room temperature in the dark and subsequently sonicated for 3 min. After centrifugation at 800 × g for 1 min, the supernatant was transferred to a new tube and the sediment was washed three times with 5 ml MilliQ water. The supernatants were pooled and filtered through 0.45 μm filters to remove larger particles and cells. Viruses were counted by flow cytometry as described below. For testing the extraction efficiency of viruses, extracted viruses were counted after the first extraction and in the supernatant after each of the washing step (Supplementary Figure S2). After the second extraction step more than 98% of the viruses were extracted compared to the sum of the extracted viruses after all extraction steps.

To determine the fraction of free cells and viruses in the porewater of the sediment, triplicates of four sediment samples were analyzed. For each sample, 25 g of sediment were centrifuged for 10 min at 100 × g by using 100-ml round-bottom tubes equipped with an inlet that separates the sediment sample from the tube bottom with a 3 μm poresize polycarbonate membrane (Millipore, MA, USA). Cells were counted by fluorescence microscopy and viruses were counted by flow cytometry as described below. The fractions of free cells and viruses in the porewater were calculated in comparison to the cell and virus numbers of bulk sediment extractions, respectively.

For enumeration of viruses and cells in the effluent of the reactors, samples were thawed and viruses and cells counted by flow cytometry with a flow cytometer BD Accuri C6 (BD Biosciences, New Jersey, USA) according to Brussaard et al. (2010). Briefly, we incubated the samples with SYBR Green I at 80°C for 10 min. Thereafter, samples were counted in the flow cytometer and analyzed based on side scatter and fluorescence intensity using the BD Accuri C6 software.

### Calculations

The fractions of free cells (*C*_*free*_) and viruses (*V*_*free*_) were calculated based on the counts of viruses (*VDC*_*PW*_) and cells (*CDC*_*PW*_) per porewater volume, the porosity (ϕ) and cell (*CDC*_*bulk*_) and virus (*VDC*_*bulk*_) counts of bulk sediment extractions, respectively.

$$\begin{array}{l} C_{free}\left[\%\right]=\frac{CDC_{PW}\;*\;\phi \;*\;100}{CDC_{bulk}}\\ V_{free}\left[\%\right]=\frac{VDC_{PW}\;*\;\phi \;*\;100}{VDC_{bulk}} \end{array}$$

Virus and cell production rates were calculated based on respective virus or cell numbers in the effluents, the retention time (*r*_*t*_), with *r*_*t*_ = *V* ϕ *f*^−1^ determined by the sediment volume (*V*), the porosity (ϕ) and the flow rate (*f*). The calculations include an average for virus counts (*VDC*_*efflux*_) and cell counts (*CDC*_*efflux*_) of the effluent, where counts from reactors that were sampled immediately after the restart of the water flow during tidal cycles as well as numbers at the start of the experiments when virus and cell numbers in the effluent had not stabilized yet (0.5–4.5 days for the respective experiments) were excluded. Net production rates of viruses (*VP*_*net*_) and cells (*CP*_*net*_) were calculated based on the following equations:

$$\begin{array}{l} VP_{net}\left[\text{viruses c}{\text{m}}^{-3}\;{\text{h}}^{-1}\right]=\frac{VDC_{efflux}\;*\;\phi }{r_{t}}\\ CP_{net}\left[\text{cells c}{\text{m}}^{-3}\;{\text{h}}^{-1}\right]=\frac{CDC_{efflux}\;*\;\phi }{r_{t}} \end{array}$$

Oxygen consumption rates were calculated from the difference between oxygen concentrations in the inflow (*C*_*in*_) and outflow (*C*_*out*_) and the retention time (*r*_*t*_). For the oxygen consumption rates, the same time intervals than for the net virus and cell production rates were used. Oxygen consumption rates (*OCR*) were calculated according to the following equation:

$$\begin{array}{l} OCR\;\left[\mu mol\;{\text{l}}^{-1}\;{\text{h}}^{-1}\right]=\frac{\left(C_{in}-\;C_{out}\right)}{r_{t}} \end{array}$$

The gross production rates of viruses (*VP*_*gross*_) and cells (*CP*_*gross*_) were calculated from the net production rates based on the fraction of free viruses (*V*_*free*)_ and cells (*C*_*free*_):

$$\begin{array}{l} VP_{gross}\;\left[\text{c}{\text{m}}^{-3}\;{\text{h}}^{-1}\right]=\frac{VP_{net}\;*\;100}{V_{free}}\\ CP_{gross}\;\left[\text{c}{\text{m}}^{-3}\;{\text{h}}^{-1}\right]=\frac{CP_{net}\;*\;100}{C_{free}} \end{array}$$

Based on the gross production rates of viruses (*VP*_*gross*_) and cells (*CP*_*gross*_) and the virus counts (*VDC*_*bulk*_) and cell counts (*CDC*_*bulk*_) in the sediment, turnover times of virus (*VT*) and prokaryotic communities (*CT*) (replacement of the standing stock of virus and cell numbers in the sediment) were calculated according to the following equations:

$$\begin{array}{l} VT\;\left[days\right]\;=\frac{VDC_{bulk}\;*\;24}{VP_{gross}\;}\\ CT\;\left[days\right]\;=\frac{CDC_{bulk}\;*\;24}{CP_{gross}\;} \end{array}$$

The virus-mediated prokaryotic turnover (*VMPT*) is a theoretical estimate on the prokaryotic turnover with the assumption that viral lysis is the sole mortality factor and was calculated based on the cell counts (*CDC*_*bulk*_) in the sediment, the gross virus production rate (*VP*_*gross*_) and the burst size (*BS* = the number of viruses released per cell lysis event):

$$\begin{array}{l} VMPT\;\left[days\right]\;=\frac{CDC_{bulk}\;*\;BS\;*\;24}{VP_{gross}} \end{array}$$

The virus-induced prokaryotic mortality (*VIPM*) determines the proportion of the total prokaryotic mortality that is caused by virus lysis and was calculated according to the following equation (Mei and Danovaro, 2004):

$$\begin{array}{l} VIPM\;\left[\%\right]=\frac{VP_{gross}\;*\;24\;*\;100}{BS\;*\;\left(\frac{1}{CT}\right)\;*\;CDC_{bulk}} \end{array}$$

### Statistical tests

To test differences between virus and cell counts and oxygen consumption in the effluent from flow-through reactors of different lengths, ANOVA statistical test was performed. Games-Howell test (*Post-hoc* analysis; *p* < 0.01) was used and is justified by different sample sizes and an unequal variance (Ruxton and Beauchamp, 2008). Statistical tests were performed with R (version 3.3.2).

## Results

### Virus and cell numbers and oxygen consumption in the effluents of the flow-through reactors during continuous water flow

**Figure 2:**
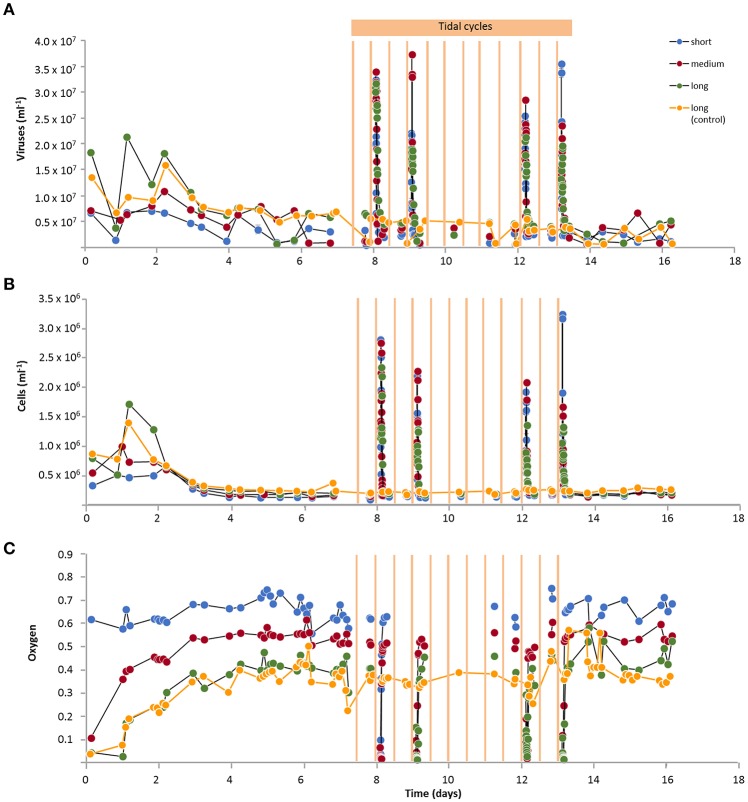
Virus and cell numbers and oxygen concentrations in effluents of sediment columns of different lengths (short, medium, 2 × long) with Janssand sediment (February) for 16 days of experimental run time. Tidal cycle simulation experiments were started after 7.5 days with three reactors, where the water flow in the reactors was stopped for 6 h followed by 6 h with continuous water flow. Tidal cycles were run twelve times in total. Only four tidal cycles (day 8, 9, 12, and 13) were sampled directly after restarting of the water flow for analyses of **(A)** virus numbers, **(B)** cell numbers, and **(C)** oxygen concentrations. Oxygen concentrations in the effluents are plotted relative to the oxygen concentrations of the reservoir. Oxygen concentrations during the tidal simulation were only measured in effluents of two reactors each time. The water flow was continuous in one long sediment column (control).

For experiments with flow-through reactors, permeable sands were filled into the reactors, seawater from a reservoir was pumped through, and virus and prokaryotic cell numbers were counted in the effluents. Virus and cell numbers in the effluents of reactors with Janssand sediment in February initially fluctuated for the first 3 days and thereafter stayed at rather constant levels during continuous water flow (Figures 2A,B; excluding increased numbers during tidal cycles). Virus numbers showed a slightly higher variation than cells. Oxygen consumption also stabilized after 3 days (Figure 2C). Similarly for experiments with Janssand sediments in June, July and August and beach sediment in November, virus and cell numbers in the effluents showed constant levels during continuous water flow after initial higher variations (Supplementary Figures S3–S6; excluding tidal cycles).

### Tidal simulation experiments

**Figure 3:**
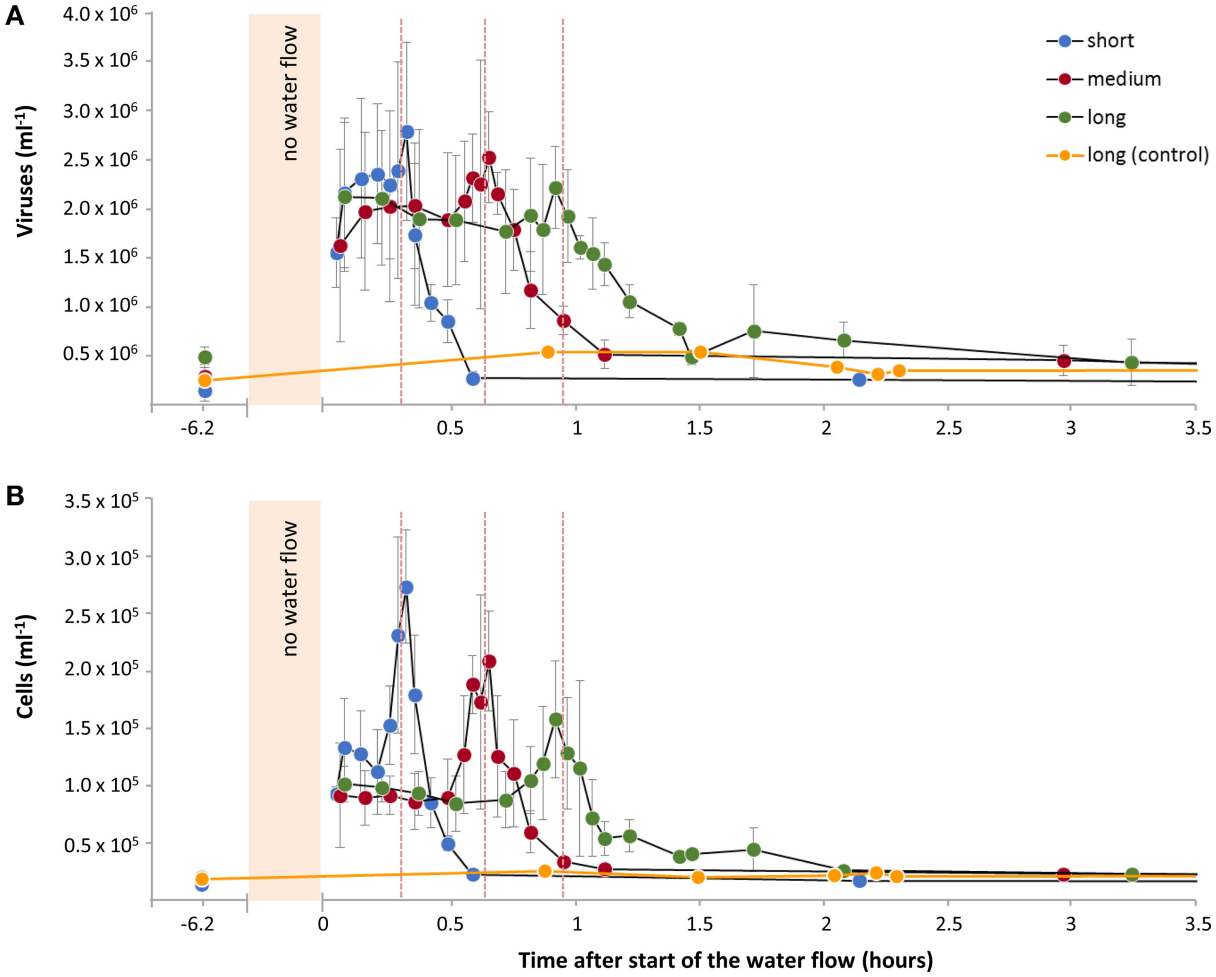
Tidal simulation experiment with Janssand sediment (February) shown in detail after restart of the water flow. **(A)** Virus and **(B)** cell numbers in effluents (averaged of four tidal cycles) analyzed in reactors with different sediment columns lengths (short, medium, long). The water flow in the reactors was stopped for 6 h followed by 6 h with continuous water flow. The water flow was continuous in one long sediment column (control). Dotted vertical lines represent the time when one porewater volume had passed through the reactors after restart of the water flow, i.e., marking the water that had been present in the sediment surface during low tide.

During tidal stimulation experiments, the water flow was halted for 6 h (hereafter called “low tide”) and subsequently restarted to continuous flow for 6 h. Out of 12 successive tidal cycles conducted during the February experiment, four cycles were sampled immediately when the water flow was restarted after low tide. Maximum virus and cell numbers in the effluents were detected after the restart of water flow (Figures 2A,B) and represent the production of viruses and cells during low tide that accumulated and were released with the effluents. In accordance to the maxima in virus and cell numbers, oxygen concentrations were depleted in the water that had been incubating in the sediment during low tide due to microbial respiration (Figure 2C). Thereafter, oxygen concentrations increased to similar levels as during continuous flow. The averaged virus and cell numbers of these four tidal cycles showed that the maxima were detected sooner (shorter retention time) when the sediment column length was shorter (Figure 3). Thus, during the first exchange of the porewater with water from the reservoir, virus and cell numbers increased to reach highest numbers in the effluent water that had been present as porewater in the surface sediment of the reactors during low tide (Figure 3; vertical dotted lines). After the first porewater exchange, virus and cell numbers decreased slowly due to mixing effects of water within the sediment and funneling of water from the sediment to the sampling port. Numbers similar to levels during the continuous flow were reached after 1.5 to 1.9 porewater volume exchanges.

Similar increases in virus and cell numbers directly after the restart of the water flow during tidal simulations were found in all experiments (Supplementary Figures S3–S6). Virus and cell numbers were 2- to 22-fold elevated directly after restart of the water flow compared to numbers during continuous water flow.

### Virus and cell numbers in surface sediments and fractions of free viruses and cells

Prokaryotic cell numbers in surface sediments were highest in Janssand sediments sampled in the summer months (June, July, August) compared to February. In sediments from Spiekeroog beach, cell numbers were 6- to 40-fold lower (Table 1). Virus counts in the sediments generally exceeded cell counts except for the beach sediment (Table 1). At the end of the experiments, virus and cell numbers of Janssand sediment from the flow-through reactors were similar to numbers at the start and showed only minor differences in the vertical distribution (Table 1). Cells in the beach sediment grew during the reactor experiments resulting in overall higher virus and cell numbers at the end of the experiment, which decreased with depth of the sediment column (Table 1). Growth was probably a result of using Janssand site water for the experiments with the beach sediment, which most likely contained more nutrients than the seawater off the beach site (Figure 1). The fraction of free viruses with respect to total virus counts in Janssand sediment was 3.2 ± 0.7%, while free cells constituted 1.4 ± 0.3% of total cell counts.

**Table 1:** Virus and cell numbers (cm^−3^) in the sediments at the start and end of the experiments.

		**Janssand June**	**Janssand July**	**Janssand February**	**Janssand August**	**Beach November**
Viruses	Start	5.2 ± 2.1 × 10^9^	4.0 ± 2.1 × 10^9^	2.5 ± 0.3 × 10^9^	4.9 ± 0.7 × 10^9^	6.9 × 10^7^
	End, top	1.8 ± 1.0 × 10^9^	nd	4.0 ± 1.4 × 10^9^	2.3 ± 1.0 × 10^9^	8.4 × 10^8^
	End, middle	5.5 ± 0.9 × 10^9^	nd	2.6 ± 0.5 × 10^9^	nd	2.6 × 10^8^
	End, bottom	4.2 ± 1.1 × 10^9^	nd	3.1 ± 1.0 × 10^9^	1.9 ± 0.4 × 10^9^	1.7 × 10^8^
Cells	Start	2.3 ± 0.1 × 10^9^	3.5 ± 0.6 × 10^9^	5.4 ± 2.2 × 10^8^	1.1 ± 0.2 × 10^9^	9.3 ± 2.6 × 10^7^
	End, top	2.6 ± 1.0 × 10^9^	2.1 ± 1.2 × 10^9^	7.2 ± 3.6 × 10^8^	4.9 ± 0.8 × 10^8^	2.2 ± 0.9 × 10^9^
	End, middle	2.5 ± 1.0 × 10^9^	nd	6.4 ± 0.9 × 10^8^	nd	5.2 ± 1.6 × 10^8^
	End, bottom	2.2 ± 0.7 × 10^9^	nd	7.5 ± 1.2 × 10^8^	1.0 ± 0.6 × 10^9^	3.7 ± 0.7 × 10^8^

*At the end of the experiments, sediment in reactors was sectioned (except for July) into top, (middle) and bottom; numbers of cells and viruses represent averages of different reactors (except virus numbers for the beach sediment). nd: not determined*.

### Net and gross production rates of viruses and cells from reactor experiments

Virus and cell numbers in the effluents represent a release from the sediments in the reactors. To show that this release represents the net production of viruses and cells in the sediment, we conducted an experiment where formaldehyde-amended seawater was pumped through the sediment in order to kill cells and to stop production of viruses and cells. In this dead control, virus and cell counts decreased exponentially with time from 1.1 × 10^6^ viruses ml^−1^ and 1.1 × 10^5^ cells ml^−1^ to 1.2 × 10^4^ viruses ml^−1^ and cell counts below detection, while the oxygen concentration in the effluent increased rapidly to a constant level of 93% compared to the reservoir with seawater (Supplementary Figure S7). The results show that when the cells in the sediment were killed, oxygen consumption decreased rapidly and both cells and viruses were washed off the sediment. In contrast in “live” sediment, numbers of cells and viruses bound to the sediment particles did not change (Table 1) and numbers in the effluents were constant during continuous flow (Figure 2). Thus, we conclude that viruses and cells in the effluent were not merely washed off the sediments but were constantly produced. While the major part of the produced viruses and cells adsorbed and decayed inside the sediment, the viruses released in the effluent represent the minimum net production. Accordingly, we calculated net virus and cell production rates as well as oxygen consumption rates from average numbers in the effluents during continuous flow for each experiment to be 0.3–13.2 × 10^6^ viruses cm^−3^ h^−1^, 0.3–10.0 × 10^5^ cells cm^−3^ h^−1^ and 56–737 μmol oxygen l^−1^ h^−1^, respectively (Table S1). Assuming that virus decay and cell mortality rates equal virus and cell production, respectively, gross production rates were estimated from the net production corrected for the relative proportion of adsorbed viruses and cells.

### Experiments with different column lengths

**Figure 4:**
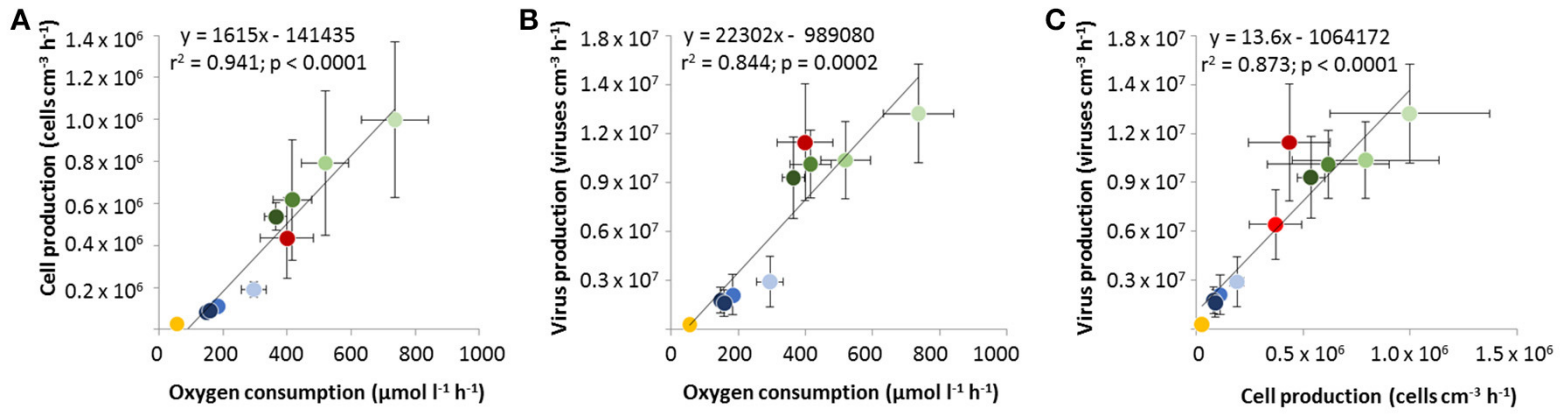
Linear correlations of net virus production rates, net cell production rates and oxygen consumption rates of experiments in reactors, respectively (see also Table S1). Significant (*p* < 0.01) correlations for **(A)** cell production rates versus oxygen consumption rates, **(B)** virus production rates versus oxygen consumption rates and, **(C)** virus production rates versus cell production rates. Yellow: experiment with beach sediment in November, blue: experiment with Janssand sediment in February (dark blue: long columns, blue: medium column, light blue: small column), dark red: experiment with Janssand sediment in July, bright red: experiment with Janssand sediment in June (note that there are no data for oxygen consumption because with this sediment the effluents of the columns were anoxic), green: experiment with Janssand sediment in August (darkest green: longest columns, dark green: long column, green: medium column, light green: small column).

In February and August, reactors were filled with sediment columns of different lengths resulting in higher virus and cell numbers (ml^−1^ effluent) as well as oxygen consumption in the effluents of reactors with longer sediment columns (Figure 2, Supplementary Figure S6). Thus, all three represented a function of sediment volume. For the February experiment, cell numbers in the effluents and oxygen consumption showed significant increases with sediment column length (*p* < 0.01), while virus numbers were significantly lower for the shortest columns in comparison to the longest columns (*p* < 0.01). For the August experiment, the four different column lengths showed significant differences for the oxygen consumption (*p* < 0.01), while for virus numbers the short, medium and long columns were significantly different and for cell numbers only the shortest column differed significantly from the other three columns (*p* < 0.01). In contrast by calculating average rates, oxygen consumption rates (μmol l^−1^ h^−1^) and net virus and cell production rates (cm^−3^ h^−1^) were higher for shorter sediment columns, i.e., smaller sediment volumes (Figure 4, Table S1). The three rates were higher during the summer months for Janssand surface sediments compared to February, while rates were lowest for the beach sediment. Virus production, cell production and oxygen consumption rates of all experiments were positively correlated (*p* < 0.01; Figure 4).

**Figure 5:**
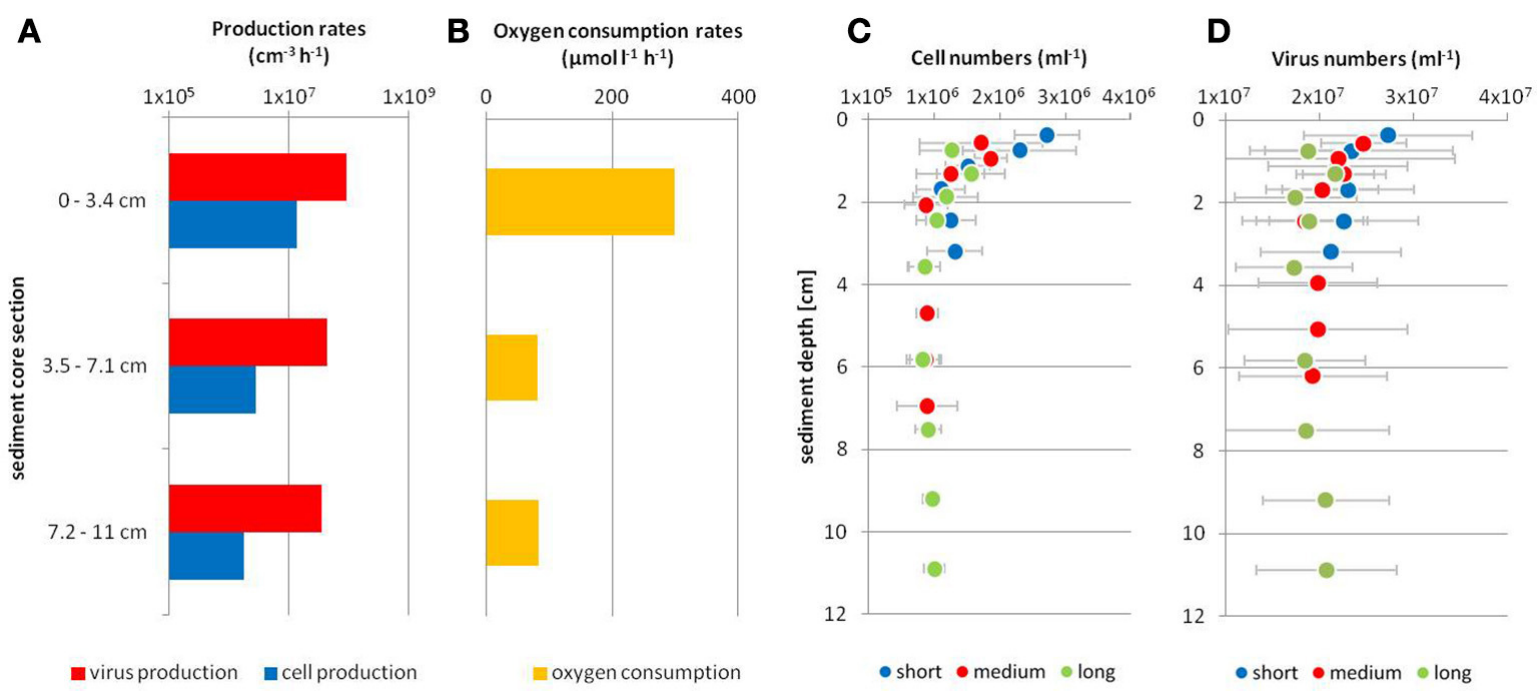
Gross virus and cell production rates, oxygen consumption rates, virus, and cell numbers in the effluents during tidal simulation experiments of the February experiment. Virus and cell production rates as well as oxygen consumption rates **(A,B)** for different column sections in the reactors were derived by the following calculations: section 0–3.4 cm values of the short column, section 3.5–7.1 cm subtraction of values from the short column from the medium column and section 7.2–11 cm subtraction of the values of the medium column from the long column during continuous water flow and the proportion of free viruses and cells in the sediments. Virus and cell numbers **(C,D)** represent numbers of the different sediment columns released in the effluents after 6 h of low tide (average of four tidal cycles, see Figure 3). Numbers are plotted at the respective column depth where the sampled water had been incubating during low tide by calculations including the porewater volume and the retention time of the porewater.

For the February experiment with three different sediment column lengths, oxygen consumption and gross virus and cell production rates were calculated for three sediment depth sections of the reactors (top, middle, bottom; Figure 5). Here, we assumed that the short column was representative for the same sediment volume at the top of both, the medium and long columns, while the medium column was representative for the same volume at the top of the long column. Gross virus and cell production as well as oxygen consumption rates were highest in the top section of the sediment columns (Figures 5A,B). While cell production rates decreased by a factor of 10 with depth, virus production rates decreased by a factor of 5 only. Based on the retention times, virus and cell numbers in the effluents (Figure 3) were transposed according to the depth where the porewater incubated in the reactors during low tide (Figures 5C,D). Accumulation of viruses and cells was highest in the surface after 6 h of low tide and decreased with depth.

## Discussion

The total number of viruses in sediments is a result of virus production and decay as well as import and export processes. In recent years the effort to measure virus production (Hewson and Fuhrman, 2003; Middelboe and Glud, 2006; Danovaro et al., 2008a) and decay (Fischer et al., 2003, 2004; Corinaldesi et al., 2010) in aquatic sediments has increased by the development of various methods (Montanié et al., 2015; Rastelli et al., 2016). In muddy and subsurface sediments, import and export of viruses are restricted to diffusion, whereas in sandy sediments both processes might contribute significantly to virus numbers as advection accelerates porewater transport and exchange between porewater and bottom water (Ahmerkamp, 2016). However, many viruses adsorb to the sediment matrix after production and during transport (Dowd et al., 1998; Ghanem et al., 2016).

This study represents the first investigation of virus production and export in permeable sediments by flow-through reactors. By quantification of the release of viruses and cells from the sediment, we were able to calculate production rates and investigate the impact of season, tidal cycles and sediment location. Estimates of gross virus production rates revealed unexpected differences along the vertical depths of the sediment column in the reactors resulting in a shift of the relative impact of viruses on the prokaryotic community with depth.

### Oxygen consumption, virus production and cell production in permeable sediments are influenced by season

Flow-through reactors have been increasingly used to study microbial processes in advection dominated sediments (Santos et al., 2012; Marchant et al., 2016; Ahmerkamp et al., 2017). Total oxygen consumption rates of 56–737 μmol l^−1^ h^−1^ (Table S1) were similar to rates measured previously in flow-through reactors with Spiekeroog beach sediments of 44–133 μmol l^−1^ h^−1^ and Janssand sediments of 271–459 μmol l^−1^ h^−1^ (Marchant et al., 2014; Beck et al., 2017). Differences in oxygen consumption rates between the experiments reflect findings that prokaryotic production and total oxygen consumption in general show a seasonal trend with higher rates during summer (Billerbeck et al., 2006; Hubas et al., 2007; Marchant et al., 2014). Seasonal differences in the environment can partly be explained by the temperature effect. As microbial communities in intertidal sediments of the German North Sea are susceptible to wide temperature fluctuations (Billerbeck et al., 2006; Beck et al., 2008b), we did not expected temperature to be a major selection factor for indigenous communities during our experiments, which were all conducted at 15°C. Furthermore, temperature appears to have only minor effect on virus production rates with increases in virus production rates at increasing temperatures (Siem-Jørgensen et al., 2008; Carreira et al., 2013). For our experiments, higher organic matter content in the seawater and sediments during the summer months was the most likely explanation for differences in cell and virus production rates and oxygen consumption (Stevens et al., 2005; Siem-Jørgensen et al., 2008).

Virus production rates are commonly measured in incubations of undiluted or diluted sediment, where the increase in virus counts is quantified over several hours (Rastelli et al., 2016). Therefore, viruses are extracted from the sediment to include viruses that are attached to sediment particles. For the experiments with flow-through reactors, virus numbers in the effluents unlikely represented the gross virus production inside the sediment columns. Instead, viruses partly absorbed to sediment grains depending on the physicochemical surface properties of the respective virus, which includes size, hydrophobicity and morphology of the virus (Ghanem et al., 2016). Moreover, sorption of viruses is also dependent on the pH of the solution, redox conditions and organic matter content of sediments (Klitzke et al., 2015). Net virus production rates derived from the flow-through reactor experiments were 0.03–1.3 × 10^7^ viruses cm^−3^ h^−1^ (Table S1). Taking the proportion of free viruses into account, we estimated gross virus production rates of 3.6–9.1 × 10^7^ viruses cm^−3^ h^−1^ for the February experiment (Figure 5). Net and gross virus production rates of our experiments are in the same range as previous measurements with the incubation technique, which determined virus production rates between 10^6^ and 10^8^ viruses cm^−3^ h^−1^ in various marine surface sediments (<30 cm) (Hewson and Fuhrman, 2003; Middelboe et al., 2006; Danovaro et al., 2008a; Siem-Jørgensen et al., 2008; Corinaldesi et al., 2010).

Virus production, cell production and oxygen consumption rates of all experiments were positively correlated (Figure 4). Oxygen consumption rates, as parameter for overall microbial activity, were linearly correlated with cell production rates indicating that higher growth rates within the sediments were a result of higher microbial activity (Figure 4A). Cells in the porewater of sandy sediments at site Janssand represent 1.4% of the total cells, whereas 0.2% free cells were found formerly for self sands of the Middle Atlantic Bight (Rusch et al., 2003). Thus, in our experiments likely only a minor part of the cells was washed out in the effluent, while the majority of the cells was firmly attached to the sand grains (Ahmerkamp, 2016). Nonetheless, the results suggest that for all experiments the proportion of cells that were free in the porewater and transported with the water flow was constant and correlated to microbial activity of total cells in the sediments. Interestingly, the virus production rates were linearly correlated with both, the oxygen consumption rates and the cell production rates (Figures 4B,C), indicating that the virus production within the reactors was correlated to activity and growth of the host cell community, which is in concert with reports for various surface sediments (Middelboe and Glud, 2006; Danovaro et al., 2008a; Siem-Jørgensen et al., 2008). Differences between the experiments likely arose from seasonal effects, as organic carbon mineralization rates have been found to vary seasonally by 7-fold for Janssand sediment in the backbarrier tidal area of Spiekeroog (Billerbeck et al., 2006), while rates at the North beach of Spiekeroog, facing the open North Sea, are generally lower (Beck et al., 2017). Thus, the flow-through reactors represent a reliable tool to study virus production in sediments with advective porewater transport and furthermore provide versatile options for experimental manipulations.

### Tidal cycles induce dynamic changes in virus and cell numbers

The flow-through reactors were started with homogenized sediments. Unexpectedly, highest average virus and cell production rates were determined for the short sediment columns compared to longer columns for the February and August experiments (Table S1). We concluded that the reason for this was a decrease in microbial activity with column depth during the experiments. This likely originated from the consumption of energy-rich and easily degradable dissolved organic matter (DOM) from the inflowing seawater by the cells in the surface sediments of the reactors. Higher nutrient input in the surface supported higher activity, while cells in the sediment below had to utilize more refractory DOM resulting in lower activities (Cowie and Hedges, 1994; Oni et al., 2015). For the experiment with beach sediment, this effect was even more pronounced. Cells grew during the experiment due to the high nutrient concentrations of the seawater, which eventually resulted in higher virus and cell numbers in the surface compared to the bottom sediment of the reactors (Table 1). This supports our hypothesis that already within the short time frame of the experiments, activity and production rates of the cells and viruses, respectively, were not evenly distributed within the reactor but decreased with depth. The results clearly showed the decrease in virus and cell production rates as well as oxygen consumption rates with depth of the sediment columns with $\frac{1}{2}$ to of the total virus production, cell production and oxygen consumption occurring in the top $\frac{1}{4}$ of the sediment (Figures 5A,B).

The differences of microbial activity in short and long columns and accordingly surface and bottom of the sediment columns were also observed during the tidal simulation experiments. Here, the maxima of virus and cell numbers were determined in the effluent that had been present as porewater in the surface sediment of each reactor during low tide (Figures 3, 5C,D), again demonstrating highest virus and cell production rates at the surface also during 6 h without water flow. After one complete flow through of the sediment with water from the reservoir, virus and cell numbers slowly decreased to similar levels as observed for the continuous water flow (Figure 3). Ultimately, viruses and cells that had been produced and accumulated in the porewater during low tide were completely washed off the sediment. In conclusion, the sediment columns in the reactor seemed to exhibit similar conditions to the natural habitat, where the microbial community readily consumes the most labile organic matter at the surface leaving less easily degradable material behind and thus, leading to the decreasing microbial activity with sediment depth (Cowie and Hedges, 1994; Oni et al., 2015).

### Decreasing virus turnover with sediment depth is opposed by an increasing virus-mediated cell turnover

The virus turnover in sediments was 1.8, 2.4, and 3.6 days for the top, middle and bottom sections of the columns for the February experiment, respectively (Figure 5). Similar virus turnover times were reported for deep-sea surface sediment of Sagami Bay, Japan, with 5 to 6 days and surface marine sediment of Øresund, Denmark, with 1 to 28 days (Glud and Middelboe, 2004; Middelboe et al., 2006; Siem-Jørgensen et al., 2008). Virus-mediated prokaryotic turnover during the February experiment ranged between 5 and 13 days for an assumed low burst size of 14 (Glud and Middelboe, 2004; Mei and Danovaro, 2004) and between 33 and 91 days for a burst size of 100 as an upper range of burst sizes determined for sediments (Hewson and Fuhrman, 2003; Danovaro et al., 2008a). As these calculations strongly depend on the burst size, also other studies estimated variable virus-mediated cell turnover of 0.3–1 days (assuming a burst size of 50–100) for sands of the Southern California Bight (Hewson and Fuhrman, 2003) and 3–45 days (assuming a burst size of 14) for coastal sediments of the Baltic Sea (Glud and Middelboe, 2004; Siem-Jørgensen et al., 2008). Overall, the activity of viruses and cells and their community turnover times in the sediment columns decreased with depth. In contrast, the relative impact of viruses on the prokaryotic community was stronger in deeper sediment layers of the reactors. Accordingly, viruses accounted for 7, 16, and 20% (assuming a burst size of 14) and 48, 113, and 144% (assuming a burst size of 100) of the prokaryotic mortality for the top, middle and bottom section of the sediment columns in February, respectively. Similarly, an increase of virus-induced mortality with depth (up to 43 and 100%) was observed for deeper sediment depths of 30 and 100 cm at two sites in the Mediterranean Sea, respectively (Mei and Danovaro, 2004; Corinaldesi et al., 2010). In conclusion, the decreasing microbial activity with depth led to longer turnover times of viruses and cells. However, viruses became more important for the prokaryotic mortality and thus the biogeochemical cycling in deeper sediments. Virus-induced cell lysis likely provides labile organic compounds for fueling prokaryotic communities in deeper sediment zones that are missing the input of fresh organic material from the water column.

### Viruses are distributed by advective transport in intertidal permeable sediments

Permeable sandy sediments allow for advective transport through their pore spaces. In intertidal sediments during high tide, advection is mainly driven by pressure gradients formed by tidal and wave pumping. This leads to rapid “skin circulation” through the surface enhancing the transport of particulate and dissolved organic matter, other nutrients and electron acceptors like oxygen several cm down into the sediments (Jansen et al., 2009). During low tide, advective transport in air-exposed surface sediments stops, while the hydraulic gradient between seawater and porewater level results in horizontal water flow in deeper layer toward the margins (“body circulation”) (Billerbeck et al., 2006; Beck et al., 2008c; Jansen et al., 2009). Furthermore, oxygen penetration is restricted and oxygen and nutrients become quickly depleted, while metabolic products of microbial processes accumulate (Rocha et al., 2005; Jansen et al., 2009). During tidal simulation experiments, oxygen depletion in the porewater was observed concomitant to the accumulation of viruses and cells in the porewater. The accumulation of viruses during 3 h of emersion of an intertidal mudflat was recently shown with *in situ* sampling of surface sediments during low tide (Montanié et al., 2015). In the absence of advection at low tide, ongoing virus production mediates a release of cell components by lysis. Our data suggests that the virus-mediated cell turnover stimulated the microbial community via the viral shunt and possibly counteracting the carbon limitation caused by the absence of advection during low tide. Furthermore, advection-driven transport distributes accumulated viruses, cell debris and metabolic products into deeper sediment layers which likely stimulate microbial activity by the introduction of labile organic carbon. Intertidal, permeable sediments represent a highly dynamic environment with an advection-driven displacement of viruses and nutrients resulting in a complex, spatial and temporal interplay of prokaryotic and viral communities.

## Author contributions

VV and TE: designed the study and wrote the manuscript; VV, LS, and TE: performed the laboratory work and data analysis.

### Conflict of interest statement

The authors declare that the research was conducted in the absence of any commercial or financial relationships that could be construed as a potential conflict of interest.
