# Supplementary material for: Virus Dynamics Are Influenced by Season, Tides and Advective Transport in Intertidal, Permeable Sediments
Source: Front Microbiol. 2017 Dec 18;8:2526. doi: 10.3389/fmicb.2017.02526 (PMC5741694; doi:10.3389/fmicb.2017.02526)
Supplement: Supplementary file 1 [file DataSheet1.pdf]

Supplementary Material

Virus dynamics are influenced by season, tides and advective transport in intertidal, permeable sediments

Verona Vandieken*, Lara Sabelhaus, Tim Engelhardt

*** Correspondence:** Verona Vandieken: vvandieken@daad-alumni.de

1 Supplementary Figures and Tables

1.1 Supplementary Tables

Table S1. Net virus and cell production rates and oxygen consumption rates calculated from the average values of the effluents during continuous water flow through the reactors (i.e., excluding start of the experiments and values directly after low tide during tidal simulation).

Rates	Janssand June (n = 3)	Janssand July (n = 4)	Janssand February (short, medium, long, long)*	Janssand August (short, medium, long, longer)*	Beach November (n = 4)
Virus production (viruses cm^{-3} h^{-1})	$6.4 \pm 2.1 \times 10^6$	$11.4 \pm 3.6 \times 10^6$	$2.9 \pm 1.6 \times 10^6$<br>$2.1 \pm 1.2 \times 10^6$<br>$1.8 \pm 0.8 \times 10^6$<br>$1.6 \pm 0.8 \times 10^6$	$13.2 \pm 3.0 \times 10^6$<br>$10.4 \pm 2.4 \times 10^6$<br>$10.1 \pm 2.1 \times 10^6$<br>$9.3 \pm 2.5 \times 10^6$	$0.3 \pm 0.1 \times 10^6$
Cell production (cells cm^{-3} h^{-1})	$3.7 \pm 1.2 \times 10^5$	$4.4 \pm 1.9 \times 10^5$	$1.9 \pm 0.4 \times 10^5$<br>$1.1 \pm 0.1 \times 10^5$<br>$0.8 \pm 0.1 \times 10^5$<br>$0.9 \pm 0.1 \times 10^5$	$10.0 \pm 3.7 \times 10^5$<br>$7.9 \pm 3.4 \times 10^5$<br>$6.2 \pm 2.9 \times 10^5$<br>$5.4 \pm 0.7 \times 10^5$	$0.3 \pm 0.04 \times 10^5$
Oxygen consumption (µmol l^{-1} h^{-1})	anoxic	400 ± 83	295 ± 39<br>183 ± 16<br>147 ± 15<br>158 ± 18	737 ± 104<br>520 ± 74<br>416 ± 61<br>365 ± 34	56 ± 14

n: number of reactors
*For the February and August experiments different sediment column lengths (short, medium, 2 x long and short, medium, long, longer, respectively) were used for the reactors.

1.2 Supplementary Figures

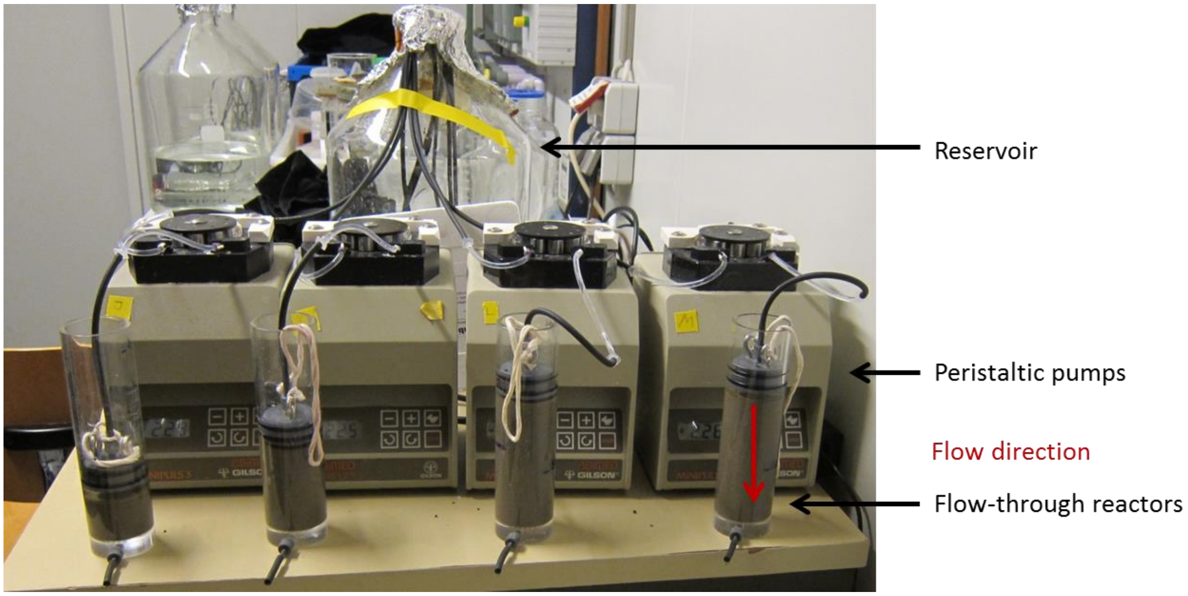

Supplementary Figure S1. Setup of the February experiment with different sediment column lengths (short, medium, long, long). From the seawater reservoir the water was pumped by peristaltic pumps on the top of the sediment columns of the flow-through reactors. For the experiment, the reservoir and the reactors were covered with black cloths.

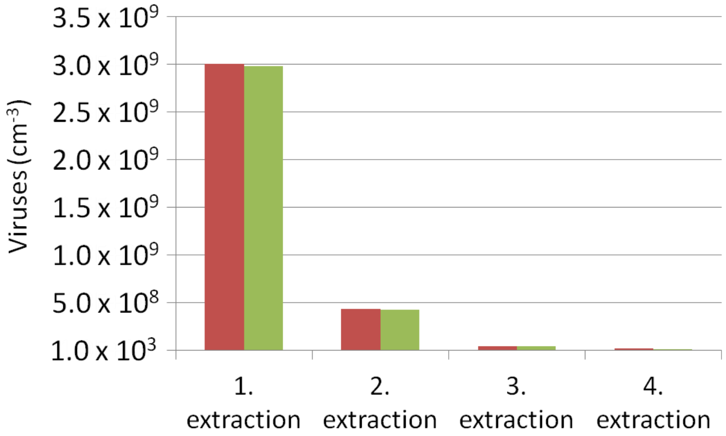

Supplementary Figure S2. Extraction efficiency of viruses from Janssand sediment. For counting, viruses were extracted from 1 cm^3 of sediment: after adding 4.5 ml 5 mM sodium pyrophosphate buffer, the extract was incubated for 15 minutes at room temperature in the dark and subsequently sonicated for 3 times for 1 minute with interruptions of 30 seconds with manual shaking. After centrifugation at 800 × g for 1 minute, the supernatant was removed and viruses were counted by flow cytometry (1st extract). The remaining sediment was washed three times by adding 5 ml MilliQ water, followed by manual shaking for 1 minute and centrifugation at 800 × g for 1 min. Viruses were counted after each washing step, representing the virus counts for the 2nd, 3rd and 4th extraction, respectively.

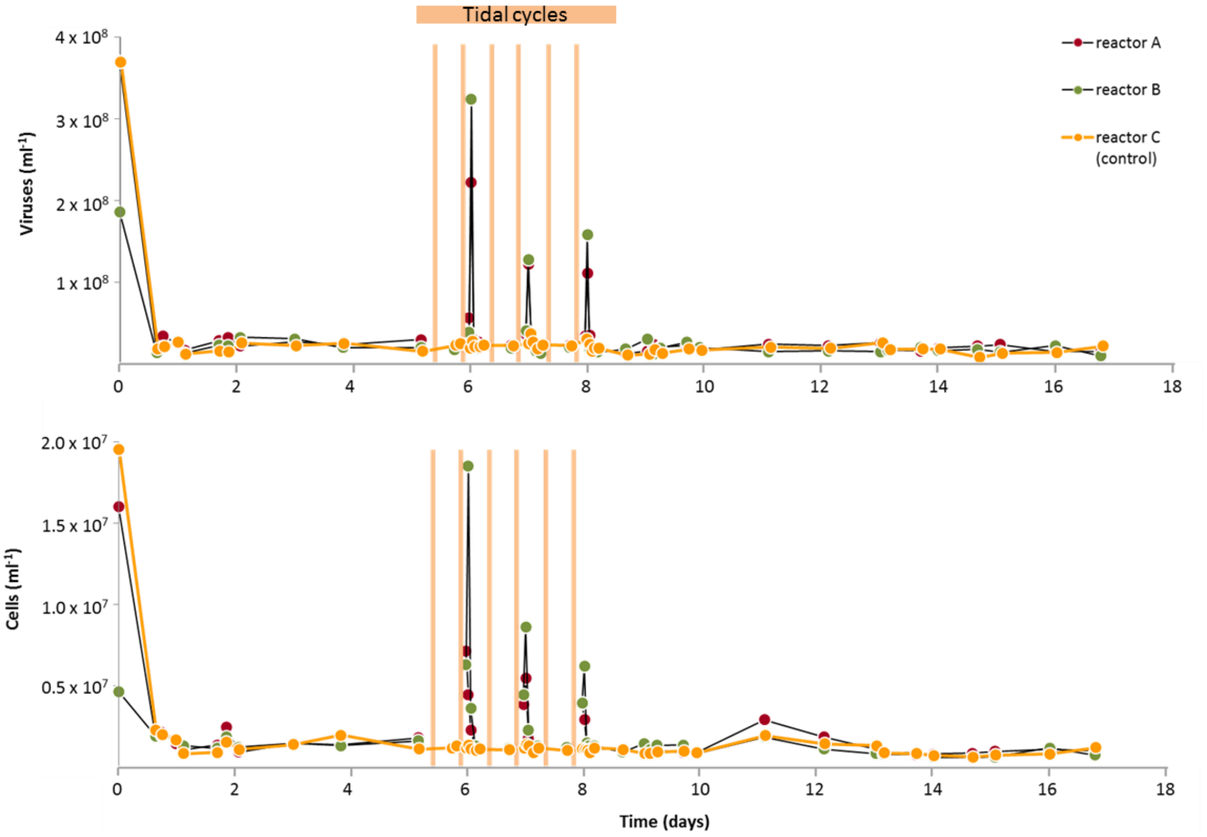

Supplementary Figure S3. Virus and cell numbers in effluents of sediment columns with Janssand sediment in June. Tidal cycle simulation experiments were started after 5.5 days with three reactors, where the water flow in the reactors was stopped for 6 hours followed by 6 hours with continuous water flow. Tidal cycles were run six times in total and only three were sampled directly after restarting of the water flow for analyses of virus and cell numbers in the effluents. The water flow was continuous in one sediment column (control).

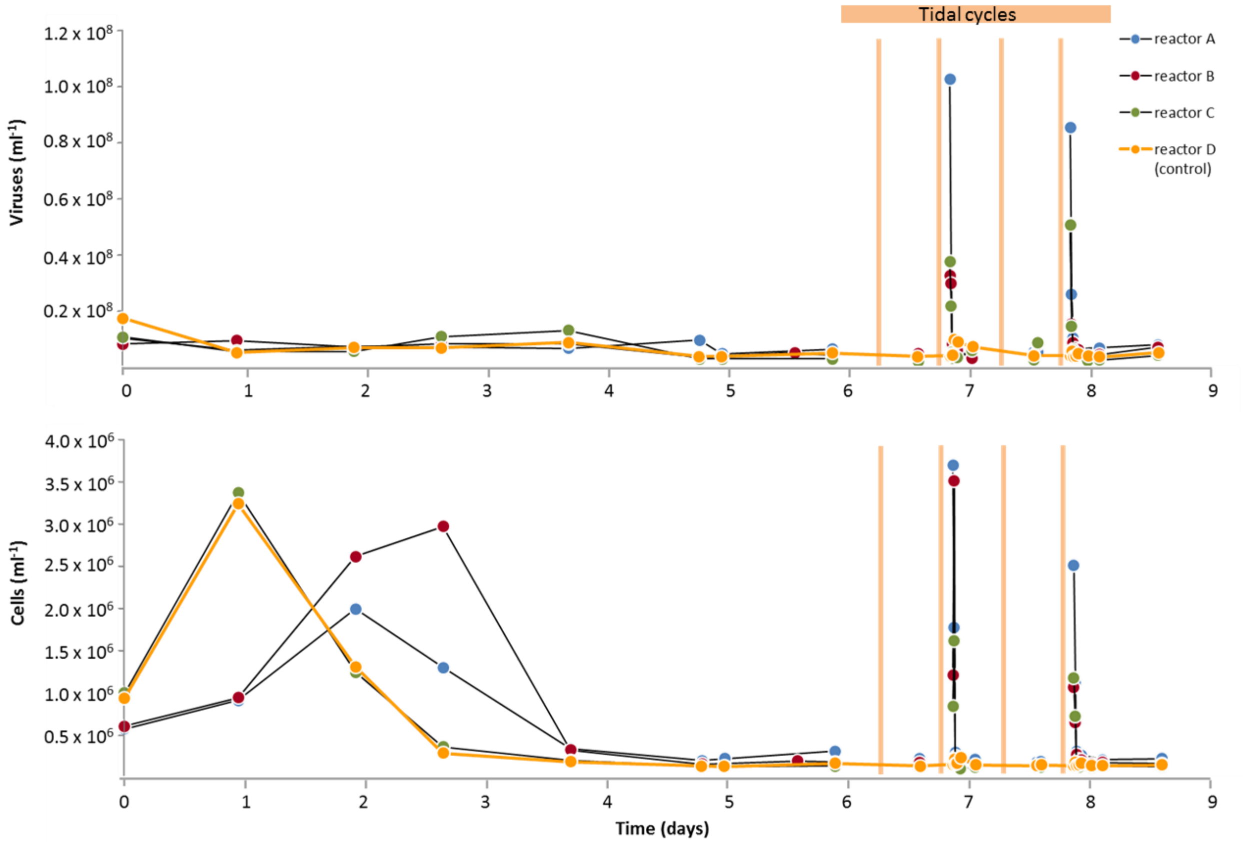

Supplementary Figure S4. Virus and cell numbers in effluents of sediment columns with beach sediment in July. Tidal cycle simulation experiments were started after 6 days with three reactors, where the water flow in the reactors was stopped for 6 hours followed by 6 hours with continuous water flow. Tidal cycles were run four times and only two were sampled directly after restarting of the water flow for analyses of virus and cell numbers in the effluents. The water flow was continuous in one sediment column (control).

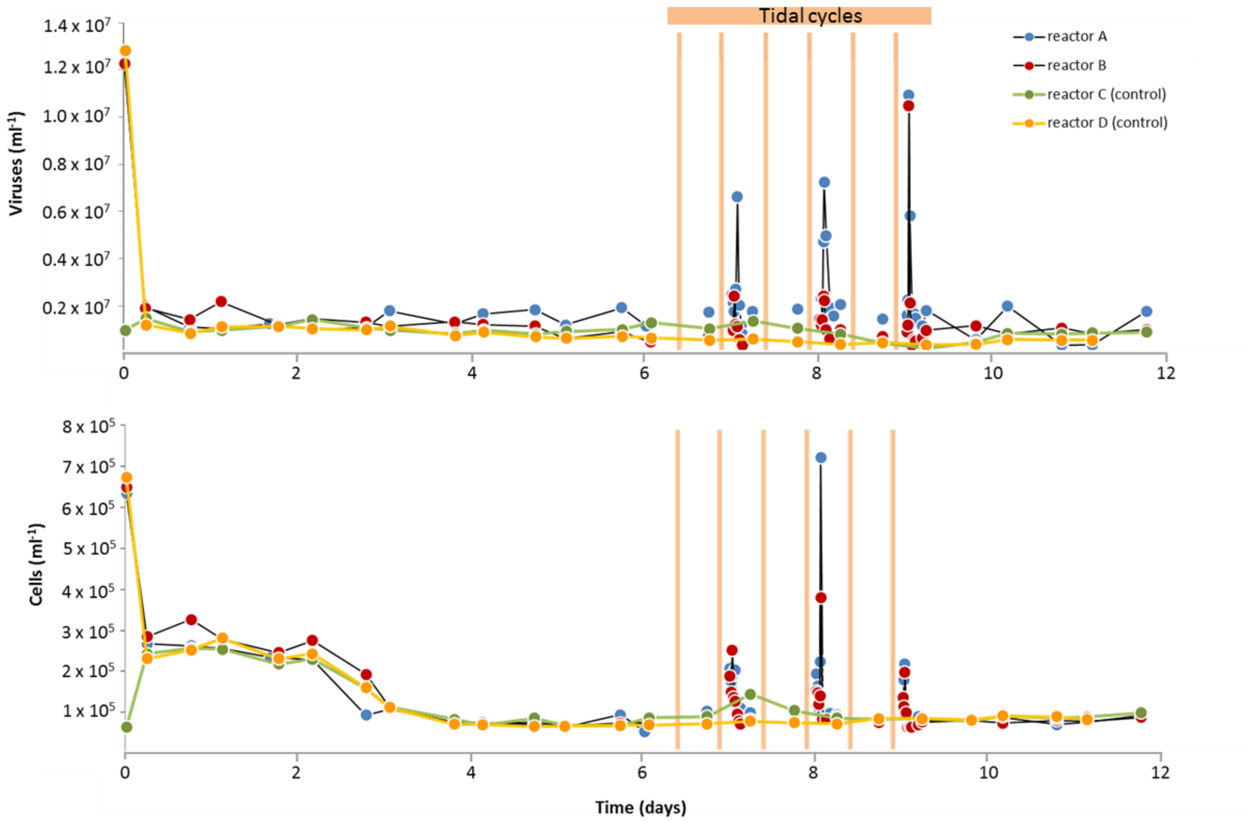

Supplementary Figure S5. Virus and cell numbers in effluents of sediment columns with beach sediment in November. Tidal cycle simulation experiments were started after 6 days with three reactors, where the water flow in the reactors was stopped for 6 hours followed by 6 hours with continuous water flow. Tidal cycles were run six times and only three were sampled directly after restarting of the water flow for analyses of virus and cell numbers in the effluents. The water flow was continuous in one sediment column (control).

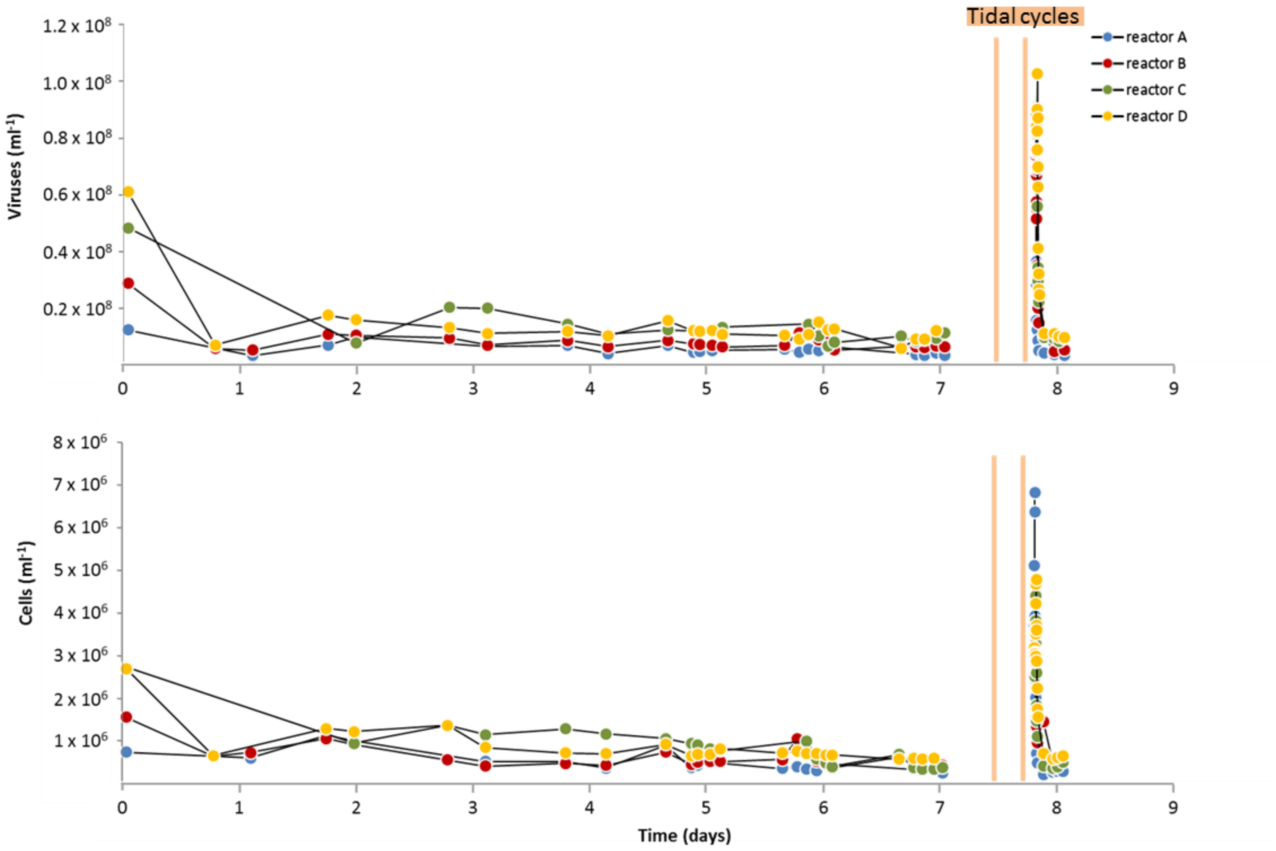

Supplementary Figure S6. Virus and cell numbers in effluents of sediment columns of different lengths: short=A, medium=B, long=C, longer=D with Janssand sediment in August. Tidal cycle simulation experiments were started after 7 days with all four reactors, where the water flow in the reactors was stopped for 6 hours followed by 6 hours with continuous water flow. Tidal cycles were run two times and only one was sampled directly after restarting of the water flow for analyses of virus and cell numbers in the effluents.

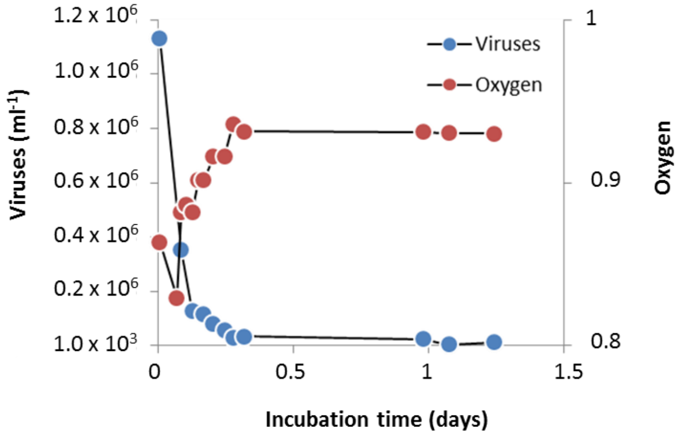

Supplementary Figure S7. Virus numbers and oxygen content in effluent of "dead" control reactor with formaldehyde-amended seawater flow through. Oxygen concentration is given as oxygen concentration in the effluent relative to the reservoir.
